# Retraction Note to: A stepwise-targeting strategy for the treatment of cerebral ischemic stroke

**DOI:** 10.1186/s12951-022-01645-w

**Published:** 2022-10-12

**Authors:** Jingbo Hu, Xueying Tan, Dongwei Wang, Yixuan Li, Hongze Liang, Jiejun Peng, Fengyan Li, Quan Zhou, Peiwu Geng, Shuanghu Wang, Yue Yu, Jin Liu

**Affiliations:** 1 Faculty of Materials Science and Chemical Engineering, Ningbo University, Ningbo, 315211 China; 2 College of Pharmacy, Zhejiang Pharmaceutical College, Ningbo, 315100 China; 3 State Key Laboratory for Managing Biotic and Chemical Threats to the Quality and Safety of Agroproducts, Institute of Plant Virology, Ningbo University, Ningbo, 315211 Zhejiang China; 4 Department of Neurosurgery, The People’s Hospital of Lishui, The Sixth Affliated Hospital of Wenzhou Medical University, Lishui, 323000 China; 5 Department of Pharmacy, Ningbo Women and Children’s Hospital, Ningbo, 315012 China

## Retraction to: Journal of Nanobiotechnology (2021) 19:371 10.1186/s12951-021-01118-6

The Editors-in-Chief have retracted this article. After publication, the following concerns were raised:

- in Fig. 3, the Heart/Saline panel and Heart/Micelle panels partially overlap, respectively, with the Heart/HA-CUR panel and Heart/CUR panel in Fig. S2 in [1];
- in Fig. 3, the Heart/Saline and Heart/Micelles panels partially overlap, respectively, with Heart/DEX-SS-IND and Heart/Saline panel in Fig. 7 in [2];
- in Fig. 4a, the TGN/cy3 micelles panel in 2.0 h line overlaps with the SA-DEX-CUR panel in the e-Selectin line in Fig. 4 in [3];
- in Fig. 5D, panels H_2_O_2_ and H_2_O_2_ + TPP-MLT overlap, respectively, with panels PTX and FA@PPMoV@PTX in Fig. 4B in [4].

Following these concerns author Jingbo Hu contacted the journal requesting a retraction. They did not respond to the editor's request to share original data. The Editors-in-Chief therefore no longer have confidence in the integrity of the data in this article.

Jingbo Hu agrees to this retraction. The other authors have not responded to any correspondence from the editor/publisher about this retraction.
